# Towards Application of One-Class Classification Methods to Medical Data

**DOI:** 10.1155/2014/730712

**Published:** 2014-03-20

**Authors:** Itziar Irigoien, Basilio Sierra, Concepción Arenas

**Affiliations:** ^1^Department of Computer Sciences and Artificial Intelligence, UPV/EHU, 20018 Donostia, Spain; ^2^Department of Statistics, UB, 08028 Barcelona, Spain

## Abstract

In the problem of one-class classification (OCC) one of the classes, the target class, has to be distinguished from all other possible objects, considered as nontargets. In many biomedical problems this situation arises, for example, in diagnosis, image based tumor recognition or analysis of electrocardiogram data. In this paper an approach to OCC based on a typicality test is experimentally compared with reference state-of-the-art OCC techniques—Gaussian, mixture of Gaussians, naive Parzen, Parzen, and support vector data description—using biomedical data sets. We evaluate the ability of the procedures using twelve experimental data sets with not necessarily continuous data. As there are few benchmark data sets for one-class classification, all data sets considered in the evaluation have multiple classes. Each class in turn is considered as the target class and the units in the other classes are considered as new units to be classified. The results of the comparison show the good performance of the typicality approach, which is available for high dimensional data; it is worth mentioning that it can be used for any kind of data (continuous, discrete, or nominal), whereas state-of-the-art approaches application is not straightforward when nominal variables are present.

## 1. Introduction

In one-class classification (OCC), the problem is to classify data when information is available for only one group of observations. Specifically, given one set of data, called the target class, the aim of the OCC methods is to distinguish data belonging to the target class from other possible classes. OCC can be seen as a special type of two-class classification problem, when data from only one class is considered. This is an interesting problem because there are many real situations where a representative set of labeled examples for the second class is too costly, difficult to obtain, or not available at all. This situation can occur, for instance, in medical diagnosis, where data from healthy or even from nonhealthy patients are extremely hard or impossible to obtain: for example, through mammograms for breast cancer detection [1, 2], the one-class recognition of cognitive brain functions [3], in prediction of protein-protein interactions [4], in the lung tissue categorization of patients affected with interstitial lung diseases [5], or in the identification of patients with one or more Nosocomial infections using clinical and other data collected during the survey [6]. Several approaches to OCC have been presented and good overviews can be found in [7–10]. Some of the OCC approaches estimate the density of the reference data and set a threshold on this density, using a Gaussian model, a mixture of Gaussians models, or the Parzen density estimators [11, 12]. Boundary methods, as the *k*-centers, NN-d [13, 14], and support vector machine SVM [15–18], cover the data set with *k* small balls with equal radii and they make assumptions about the clustering characteristics of the data or their distribution in subspaces. These methods only achieve good results when the target data have the same distribution tendency in all orientations [19]. The reconstruction methods (*k*-mean clustering, self-organizing maps, PCA, mixtures of PCAs, and diabolo networks density) make assumptions about the clustering characteristics of the data or their distribution in subspaces, and a set of prototypes is needed (see, e.g., [20]). Many of these methods have data-specific parameters or assume that data follow a specific model; therefore data knowledge is necessary. One-class classification can also be considered as outlier detection, where the classification model can be used to detect the units deviating significantly from the target class. There are some distance-based outlier detection methods [21, 22], which need the computation of the distances between units in the target class and distances between a new unit and their neighbors in the target class, but in contrast with other OCC methods they are more flexible. Some other state-of-the-art methods are neural networks [23], Bayesian neural networks [24], or Naive Bayesian classifiers [25]. Recently, in [26] the authors formulated a typicality test and this approach is here applied to the OCC problem. Thus, objects in the target class can be considered as typical, while objects in the negative class can be considered as atypical. In order to evaluate the viability of the typicality approach a comparative study is presented. Five reference state-of-the-art techniques, two parametric density methods, the Gaussian and mixture of Gaussians procedures; two nonparametric density methods, the Parzen and naive Parzen procedures; a boundary method, the support vector data description method, are experimentally compared with the typicality approach using biomedical data sets.

The paper is organized as follows. Section 2 presents the six considered OCC procedures that are evaluated for twelve real biomedical data set. The experimental study is summarized in Section 3, while conclusions are drawn in Section 4.

## 2. One-Class Classification

In this section, the one-class classification problem is formally stated, and the six considered procedures are reviewed.

### 2.1. The One-Class Classification Problem

Consider a class *C*, the target class, containing *n* objects and represented by a *S*-random vector **Y** with probability density function *f* with respect to a suitable measure *λ*. Let an object of *C* be represented by a vector **y** containing the values of the measures in *p* features, not necessarily continuous. The OCC problem can be defined as the problem of assigning or not a new object **y**_0_ to the target class *C*, when data only from the target class is available. Thus, from data in the target class a classification model should be constructed. The OCC procedures usually consider a training phase using the so-called training data set; that is, either the probability density function or the parameters of the classifier's model should be determined. In OCC the training data set contains only the observations belonging to *C*, while the testing data set includes the observations from class *C* and other possible class *C*′. As in medical care correct diagnosis is very important, it is necessary to evaluate the OCC models, which can be considered as a case/noncase diagnosis where the target class is, for instance, the case class. This diagnosis will misclassify some cases as noncases and some noncases as cases. These two types of misclassifications lead to two important aspects of the performance of the diagnosis, sensitivity, and specificity. As it is known, the sensitivity or true positive rate is the probability that occurs if an object in class *C* is classified as belonging to this class. The specificity or true negative rate is the probability that occurs if an object not belonging to *C* is classified as not belonging to *C*. A very common way of displaying the values of the sensitivity and specificity is by the ROC curve (Receiver Operating Characteristic), which represents the pairs (1-specificity, sensitivity). Therefore, the area under the ROC curve, the AUC, lies between 0 and 1 and takes value 1 for a perfect diagnosis and the value 0.5 for random diagnosis, so that AUC values will be useful to evaluate the performance of OCC models [27].

It is important to note that in one-class classifiers the ability to learn the true characteristics of the data in presence of noise or errors in the feature values is specially important. Furthermore, the number of parameters to be estimated by users should be minimized, and the computational and storage requirements must be in consideration, as there are limiting factors in the use of some of the methods. Finally, one-class classifiers are determined in the training phase using the training data set; thus the standard OCC procedures may be affected by initial settings.

Next, six one-class classification methods will be reviewed. We consider five well known and reference OCC methods: two parametric density methods, the Gaussian and mixture of Gaussians; two nonparametric density methods, the Parzen and naive Parzen; a boundary method, the support vector data description. For these methods, we summarized some of their characteristics and references for more details about the construction of the classification model and properties are given. Finally, a nonparametric typicality approach based on distances is considered. As this method has not yet been considered as a one-class classification procedure, more details about the classification model and properties will be included.

### 2.2. Gaussian and Mixture of Gaussians

The Gaussian and mixture of Gaussians methods assume that the data is distributed according to the normal distribution or to a mixture of *n*_*G*_ normal distributions [9]. The parameters of the Gaussian model can be found by maximizing the likelihood function over the training data set, being the learning process computationally inexpensive. For the mixture of Gaussians, the parameters can be found efficiently by the EM algorithm. Thus, the learning process using the EM algorithm is more computationally demanding as a number of interactions should be done before the algorithm converges. The methods based on Gaussian models are sensitive to the noise in the training data set, as the noise introduces a significant bias to the estimate covariance matrix. Furthermore, these procedures present a rather high sensitivity to errors in feature values and outliers. In the learning phase, the storage requirements are rather high but very low in the classification phase.

### 2.3. Parzen and Naive Parzen

Parzen and naive Parzen density estimation are nonparametric procedures and do not need any assumption about the data distribution [6, 28, 29]. The density is estimated directly from the training data and is a function of the number of objects situated in a region of a specific volume with a value *h* as the length of an edge. The value of *h* plays the role of a smoothing parameter. An advantage of the method is that it does not need any estimation of parameters. However, too long values of the smoothing parameter *h* imply an oversmoothed estimated density. When *h* is too small then the estimated density contains noise. Furthermore, the method needs to store all the observation vectors and it makes it slower, presenting very low computational requirements of learning but rather high in classification. The method is relatively robust to the outliers in the training data, choosing appropriate distance, and presents rather high sensitivity to errors in feature values. These procedures need to estimate one parameter by the users.

### 2.4. One-Class Support Vector Data Description

Support vector data description (SVDD) is a boundary method [9, 17]. It defines a hypersphere with a minimum volume covering the entire training data set. The minimization is solved as a quadratic programming problem and can be solved efficiently by introducing Lagrange multipliers [30, 31]. The method is relatively resistant to noise. The number of parameters that are to be estimated is equal to the size of the training data set; thus it is not useful for large training data sets. SVDD presents rather low sensitivity to errors in feature values and outliers. The method presents very high computational requirements of learning but very low in classification and needs to estimate one parameter by the user and learnt the other parameters.

### 2.5. Typicality Approach

Consider a target class *C* containing *n* units measured on *p* features. Let *δ*(**y**, **y**′) be a distance [32] function on *S*. It is said that *δ* is an Euclidean distance function if the metric space (*S*, *δ*) can be embedded in an Euclidean space *R* ^*q*^, Ψ : *ℛ* → *R* ^*q*^, such that *δ* ^2^(**y**, **y**′) = ||Ψ(**y**)−Ψ(**y**′)||^2^, and we may understand *E*(Ψ(**Y**)) as the *δ*-mean of **Y**. There are various ways of achieving this situation, the most common probably being classical metric scaling, also known as principal coordinate analysis [33, 34]. Given the real-valued coordinates **Z** = Ψ(**Y**), it is possible to apply any standard multivariate technique. Such an approach was used by different authors [35–42]. In this context a general measure of dispersion of **Y**, the geometric variability *V*_*δ*_ of *C*, with respect to *δ* can be defined by

(1) $$\begin{array}{c} V_{\delta }\left(C\right)=\frac{1}{2}\overset{}{\underset{S\times S}{\int }}\delta^{2}\left(y_{i},y_{j}\right)f\left(y_{i}\right)f\left(y_{j}\right)\lambda \left(dy_{i}\right)\lambda \left(dy_{j}\right) \end{array}$$

which is a variant of Rao's diversity coefficient [43]. The proximity function of a unit **y**_0_ to *C* is defined as

(2) $$\begin{array}{c} \phi^{2}\left(y_{0},C\right)=\overset{}{\underset{S}{\int }}\delta^{2}\left(y_{0},y_{j}\right)f\left(y_{j}\right)\lambda \left(dy_{j}\right)-V_{\delta }\left(C\right). \end{array}$$

In applied problems, the distance function is a datum, but the probability distribution for the population is unknown. Natural estimators given a sample **y**_1_,…, **y**_*n*_ coming from *C* are

(3) $$\begin{array}{c} {\hat{V}}_{\delta }\left(C\right)=\frac{1}{2n^{2}}\overset{}{\underset{i,j}{\sum }}\delta^{2}\left(y_{i},y_{j}\right),\\ {\hat{\phi }}^{2}\left(y_{0},C\right)=\frac{1}{n}\overset{}{\underset{i}{\sum }}\delta^{2}\left(y_{0},y_{i}\right)-{\hat{V}}_{\delta }\left(C\right), \end{array}$$

for the geometric variability of *C* and the proximity function of unit **y**_0_ to *C*, respectively.

See [44] and references therein for a review of these concepts, their application, different properties, and proofs.

Let **y**_0_ be a new observation and consider the OCC problem to decide whether **y**_0_ belongs to the target class *C* or, on the contrary, it is an outlier or an atypical observation, belonging to some different and unknown class. Therefore, the OCC problem can be formulated as a hypothesis test with

 
*H*_0_: **y**_0_  comes from the target class *C* with  *δ*-mean  *E*(Ψ(**Y**)),
 
*H*_1_: **y**_0_  comes from another unknown class.

This test can be considered as a test of typicality, as is formulated in [26]. In our context, with only one known class, the typicality test reduces to compute *ϕ* ^2^(**y**_0_). If *ϕ* ^2^(**y**_0_) is significant it means that **y**_0_ comes from a different and unknown class.

Sampling distribution of *ϕ* ^2^(**y**_0_) can be difficult to find for mixed data, but nevertheless it can be obtained by resampling methods, in particular drawing bootstrap samples: draw *N* units **y** with replacement from *C* and calculate the corresponding *ϕ* ^2^(**y**) values; repeat this process 10P times, with *P* ≥ 1. In this way, the bootstrap distribution under *H*_0_ is obtained.

It is worth to point out that this procedure can be used for any kind of data (continuous, discrete, or nominal), whereas other approaches application is not straightforward when nominal variables are present. As the procedure needs the computation of the distances between units in the target class and distances between a new unit and the units in the target class the storage requirements are rather high but very low in the classification phase. The method is relatively robust to the outliers in the training data.

## 3. Results of the Experimental Study

As there are few benchmark data sets for OCC, we use data sets containing two or multiple classes. Each class in turn is considered as the target class and the units in the other classes are considered as new units to be classified. On the one hand, we used 10 biomedical data sets, none of them containing nominal variables, from the UCI machine learning repository [45] to evaluate the performance of all the above procedures. In order to perform the comparison, the selected data sets are the biomedical data used in [46]; only the target classes considered in that referred work are taken into account in this paper as well. On the other hand, we also applied the typicality approach to two data sets with mixed variables.

In our experiments, we followed the procedure stated in [46]. Thus, all multiclass problems are transformed to one-class classification problems by setting a chosen class as a target class and all remaining classes as nontargets. The target class was randomly split into equal parts between the training and test sets. All one-class classifiers were only trained on the target data, that is, the half of the target data, and tested on the test data, the remaining half of the target data and the nontarget data. The experiments were repeated 10 times and the AUC average and standard deviation values are reported.

### 3.1. Results of Data Sets without Nominal Variables

In Table 1, a brief description of these well known data sets is presented. For the typicality method, a suitable distance is selected for each data set, according to the type of data (see Table 2 last column). The considered distances were the Euclidean distance, the Euclidean distance after standardized the data, the Mahalanobis distance, or the correlation distance.

With* breast Wisconsin prognostic*,* E. coli*,* hepatitis,* and* liver disorders* data sets, the typicality model obtained similar AUC average values than the other procedures, as we can see in Table 2. For the* breast Wisconsin origin* data set and taking benign class as target class, the typicality procedure obtained very good results (99.4 ± 0.2) and similar to the obtained by the other procedures. It is worth noting that, for the malignant class as target class, it obtained similar results (97.6 ± 0.5) than the naive Parzen procedure (96.5 ± 0.4) and much better results than the obtained for the other procedures. With the* colon* data set, while mixture of Gaussians is not available and Parzen or SVDD methods gave poor results with high variability (63.6 ± 22.4, 36.4 ± 22.4 for classes 1 and 2, resp.), the typicality method obtained clearly better results (75.4 ± 6.3, 78.3 ± 5.8 for classes 1 and 2, resp.) than Gaussian (61.1 ± 3.8, 70.4 ± 1.1 for class 1 and 2, resp.) or naive Parzen (73.4 ± 3.1, 70.0 ± 1.5 for classes 1 and 2, resp.) methods. When the* leukemia* data set was analyzed, mixture of Gaussians and Parzen procedures were not available at all, and SVDD procedure presented a large variability (58.9 ± 30.2 and 41.1 ± 30.2, resp.). However, similar results were found for Gaussian, naive Parzen, or typicality procedures. For the* METAS* data set, it must point out that when the second class was the target class, the best results were obtained with the typicality procedure (64.5 ± 4.7), showing its good performance with high dimensional data sets. With the* SPECT heart* data set and using the typicality method, a little worse results were found when* class 0* was the target class. However, when the target class was* class 1*, clearly the typicality procedure obtained the best results (69.8 ± 2.5). Finally, for the* thyroid* data set, the typicality results were similar or slightly better than those obtained by the other procedures.

**Figure 1:**
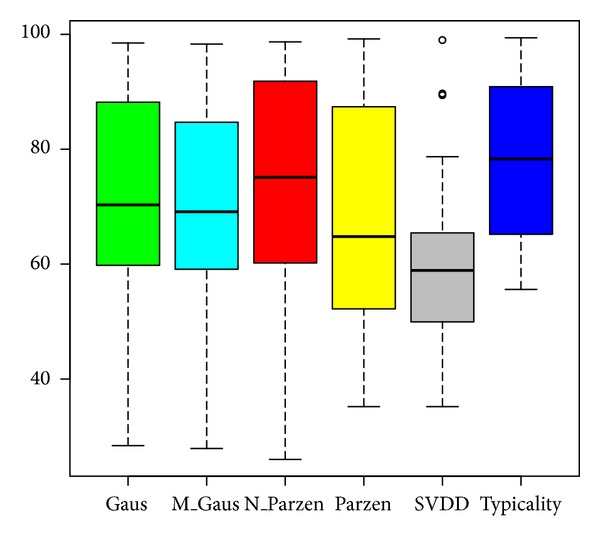
Boxplots of AUC average values of the OCC methods over the experimental data sets.

In summary, from the results presented in Table 2 it is clear that, in general, the typicality approach performs equal or better than the other well known procedures, for all the considered UCI data sets. The results show that, while other procedures are affected by small target classes, the typicality approach is more robust. Furthermore, it performs well with high-dimensional data. On the other hand, as shown in Table 2, state-of-the-art algorithms give “NaN”—Not a Number—in some cases; this fact does not appear when the typicality approach is used. Additional statistics on the AUC average values are provided in Figure 1 under the form of boxplots. Black lines correspond to the median values and black segments to the minimum and maximum values of each method. As we can see, the typicality procedure is the more robust for all data sets and it is in the top best methods.

### 3.2. Results on Mixed Variables Data Sets

Next we report the results obtained using two data sets with mixed variables. That means that there are some quantitative, binary, and nominal variables. Therefore, methods that implicitly are based on the Euclidean distance are not adequate. Thus, only the typicality approach was performed with these two data sets. In presence of mixed variables, it is known that Gower's distance is an appropriate distance, presenting good properties in terms of missing values [47, 48]. 

*Statlog (Heart) Data Set.* This data set is available in the UCI dataset repository. It is composed by 270 units classified in two classes:* absence* or* presence* of heart disease, with 150 and 120 units, respectively. There are 13 variables, 6 quantitative, 1 ordered, 3 binary, and 3 nominal, and no missing values are present. Taking in turn,* absence* and* presence* class as the target class, the typicality approach reported AUC average and standard deviation values 86.08 ± 2.03 and 84.53 ± 1.62, respectively. Furthermore, Table 3 reports the results obtained when we attempt to achieve a fixed False Alarm Rate (FAR) or false negative rate (1-sensitivity), namely, 0.1. Note that for the two target class, we obtain good results. 

*Liver Cancer Data Set.* We apply the typicality approach to a liver cancer data set [49]. It consists of 213 cases described by 4 nominal variables (type of hepatitis, categorized age, sex, and whether cirrhosis is present) plus 1993 genes. It is worth to mention that for each case at least one missing value is present (9.6% of the values are missing). The data set is divided in three groups. Group T formed by 107 samples from tumors on liver cancer patients, group NT formed by 76 samples from nontumor tissues of liver cancer patients and group N formed by 30 samples from normal livers. In [42] it was shown that there exists a high degree of confusion between groups, so bad one-class classification results are expected. Taking groups N, NT, and T as target classes, the typicality approach obtained AUC average values and standard deviation values 86.04 ± 3.95, 80.86 ± 3.07, and 55.62 ± 3.76, respectively. Results obtained for a fixed FAR equal to 0.1 are reported in Table 4. From Table 4, we can observe that when T is the target class, the method cannot distinguish the other groups. When NT is the target class, units from N group are not distinguished and only half the units from T are distinguished properly as nontarget. When N is the target class, units from T are very well distinguished as nontarget.

## 4. Conclusions

A noticeable attention has been devoted to the one-class classification problem in the last years. This type of classification is characterized by the use of observations belonging to only one known class. These methods are particularly useful in biomedical studies, when observations belonging to other classes are difficult or impossible to obtain. In this paper, reference state-of-the art one-class classification methods have been reviewed, and their suitability has been compared with a recent typicality procedure. To assess the efficiency of this new typicality application, experiments have been conducted on several public data sets from the UCI repository and has been compared to five of the most OCC used procedures, namely, Gaussian, mixture of Gaussians, naive Parzen, Parzen, and support vector DD models [46]. The results show that the typicality approach performs equally well or better than these state-of-the art procedures, thus it will be very valuable in many biomedical applications. The typicality approach does not need any knowledge about the data distribution, does not estimate any parameter, and is applicable to any kind of data, not necessarily continuous. This approach performs well with high dimensional data and it is robust in front of small target classes, whereas other OCC method accuracy rates are not so stable. For all these reasons, the typicality approach can be very useful in many biomedical applications where clinical, pathological, or biological noncontinuous data can be found and where data from healthy or even from nonhealthy patients are extremely hard or impossible to obtain.

## Figures and Tables

**Table 1:** Description of ten UCI data sets used in the experiments.

Data sets	Classes	Instances	Features
Breast Wisconsin original	2	241/458	9
Breast Wisconsin prognostic	2	47/151	33
Colon	2	40/22	1908
*E. coli *	2	52/284	8
Hepatitis	2	123/32	19
Leukemia	2	47/25	3571
Liver disorders	2	145/200	6
METAS	2	46/99	4919
SPECT heart	2	95/254	44
Thyroid	3	93/191/3488	21

**Table 2:** AUC average and standard deviation, in brackets, values on UCI data sets. In the last column the distance used by the typicality method is indicated: c: correlation, E: Euclidean, E-st: Euclidean after standardization, and M: Mahalanobis.

Data sets	Target class	Gaussian	Mixture Gaussians	Naive Parzen	Parzen	Support vector DD	Typicality distance
Breast Wisconsin original	Benign	98.5 (0.1)	98.3 (0.2)	98.7 (0.1)	99.2 (0.1)	99.0 (0.1)	99.4 (0.2)—E
	Malignant	82.3 (0.2)	69.1 (3.2)	96.5 (0.4)	72.3 (0.5)	66.1 (0.8)	97.6 (0.5)—E
Breast Wisconsin prognostic	Returning	63.0 (1.4)	59.1 (1.6)	59.0 (1.9)	59.4 (1.9)	59.6 (1.4)	58.5 (5.4)—M
	Nonreturning	50.8 (0.8)	52.6 (1.6)	53.8 (2.2)	52.2 (1.7)	51.7 (1.7)	55.6 (2.9)—M
Colon	1	61.1 (3.8)	NaN	73.4 (3.1)	63.6 (22.4)	63.6 (22.4)	75.4 (6.3)—c
	2	70.4 (1.1)	NaN	70.0 (1.5)	36.4 (22.4)	36.4 (22.4)	78.3 (5.8)—c
*E. coli *	Periplasm	92.9 (0.3)	92.0 (0.4)	93.0 (0.8)	92.2 (0.4)	89.4 (0.8)	95.4 (1.3)—E
Hepatitis	Normal	82.1 (1.0)	78.3 (1.0)	80.1 (0.7)	79.0 (1.0)	78.7 (1.1)	80.8 (2.2)—M
Leukemia	1	92.1 (1.8)	NaN	90.2 (4.4)	NaN	58.9 (30.2)	91.2 (3.4)—c
	2	94.7 (2.7)	NaN	96.7 (0.4)	NaN	41.1 (30.2)	90.6 (3.9)—c
Liver disorders	Class 1	58.5 (0.4)	59.3 (0.7)	61.4 (0.7)	58.7 (0.4)	59.0 (0.9)	58.1 (2.5)—M
	Class 2	50.9 (0.5)	49.4 (0.6)	48.4 (0.8)	46.9 (0.8)	49.6 (1.0)	58.0 (3.7)—M
METAS	1	69.1 (1.5)	NaN	65.3 (0.8)	64.8 (21.5)	64.8 (21.5)	67.3 (2.3)—c
	2	36.4 (1.4)	NaN	40.7 (1.2)	35.2 (21.5)	35.2 (21.5)	64.5 (4.7)—c
SPECT heart	Class 0	93.4 (0.9)	95.1 (0.8)	90.7 (1.5)	95.7 (1.0)	89.7 (3.2)	86.1 (3.8)—M
	Class 1	28.4 (0.5)	27.9 (1.3)	26.0 (0.7)	44.5 (0.5)	57.1 (11.1)	69.8 (2.5)—M
Thyroid	Normal	84.3 (0.0)	84.7 (4.4)	96.1 (0.0)	90.6 (0.0)	56.0 (0.0)	98.1 (1.2)—c
	Hyperthyroid	70.3 (0.0)	68.1 (0.9)	75.1 (0.0)	70.6 (0.0)	45.7 (0.0)	65.9 (2.5)—c
	Subnormal	69.6 (0.0)	81.5 (1.0)	84.4 (0.0)	87.4 (0.0)	50.3 (0.0)	88.0 (2.8)—c

**Table 3:** For Statlog (heart) data set and using the typicality approach, false and true positive, and negative values, for a fixed False Alarm Rate equal to 0.1.

Target class	Classified as	Tested classes	
		Absence	Presence
Absence	Target	135/150	41/120
	Nontarget	15/150	79/120

Target class	Classified as	Tested classes	
		Presence	Absence

Presence	Target	110/120	68/150
	Nontarget	10/120	82/150

**Table 4:** For Liver cancer data set and using the typicality approach, false and true positive, and negative values, for a fixed False Alarm Rate equal to 0.1.

Target class	Classified as	Tested classes		
		N	NT	T
N	Target	29/30	56/76	16/107
	Nontarget	1/30	20/76	91/107

Target class	Classified as	Tested classes		
		NT	N	T

NT	Target	72/76	28/30	48/107
	Nontarget	4/76	2/30	59/107

Target class	Classified as	Tested classes		
		T	N	NT

T	Target	102/105	30/30	75/76
	Nontarget	3/105	0/30	1/76
